# Draft Genome Sequence of Uropathogenic *Escherichia coli* Strain NB8

**DOI:** 10.1128/genomeA.00944-16

**Published:** 2016-09-08

**Authors:** Xing-bei Weng, Zu-huang Mi, Chun-xin Wang, Jian-ming Zhu

**Affiliations:** a Department of Medical Laboratory, Ningbo First Hospital, Ningbo, China; b Department of Bioinformation, Wuxi Clon-Gen Technology Institute, Wuxi, China; c Department of Medical Laboratory, Wuxi People's Hospital affiliated with Nanjing Medical University, Wuxi, China; d Department of Medical Laboratory, Hangzhou Yuhang Hospital of Traditional Chinese Medicine, Hangzhou, China

## Abstract

*Escherichia coli* NB8 is a clinical pyelonephritis isolate. Here, we report the draft genome sequence of uropathogenic *E. coli* NB8, which contains drug resistance genes encoding resistance to beta-lactams, aminoglycosides, quinolones, macrolides, colistin, sulfonamide-trimethoprim, and tetracycline. NB8 infects the kidney and bladder, making it an important tool for studying *E. coli* pathogenesis.

## GENOME ANNOUNCEMENT

Urinary tract infections (UTIs) account for more than 7 million office visits and 1 million hospitalizations annually in the United States, making them the most common bacterial infections acquired in the community and in hospitals (1). Uropathogenic *Escherichia coli* is the predominant species, leading to 75% to 95% of UTIs in otherwise healthy young women (2). Adhesins, siderophores, and toxins enable UPEC strains to colonize and invade the urinary tract (3). Treatment of UTIs with antibiotics selects for resistant uropathogens, and uropathogens are increasingly becoming resistant to currently available antibiotics. In addition, UTIs often reoccur, and recurrent UTIs further lead to high antibiotic usage (3). UPEC strain NB8 is a clinical pyelonephritis isolate, and the variety of virulence genes and resistance genes in the genome sequence of NB8 will thus serve as a useful resource for future studies into bacterial survival, antibiotic resistance, and recurrent UTIs of this important human pathogen. NB8 genomic DNA was sequenced on the Ion Torrent (200-bp reads) and the Illumina HiSeq platform (100-bp paired-end reads), according to the manufacturers’ protocol. The reads were quality filtered and assembled in two phases. *De novo* assembly was done with Edena version 3 (4), Assembly by Short Sequences (ABySS) version 1.3.1 (5), and Velvet version 1.2 (6). Reads were also aligned to *Escherichia coli* SE15 (GenBank accession no. NC_013654). The *de novo* assemblies and alignment-based contigs were merged using Gap4 (7), scaffolded with SSAKE-based Scaffolding of Pre-Assembled Contigs after Extension (SSPACE) version 2.0 (8), and gaps were PCR amplified and sequenced by Sanger sequencing. The assembly of a pseudochromosome resulted in 14 contigs, and unmapped contigs resulted in 33 contigs, which were annotated via the NCBI Prokaryotic Genome Annotation Pipeline (PGAP) (9). The pseudochromosome is 4.55 Mb, and its G+C content is 50.17%. The unmapped contigs are 635 kb, and their G+C content is 48.56%. NB8 provides a total of 5,248 genes, 5,023 coding sequences (CDSs), 18 rRNAs (5S, 16S, and 23S), 85 tRNAs, 13 noncoding RNAs (ncRNAs), and 62 frameshifted genes.

*In silico* genome analysis identified 17 antimicrobial resistance genes encoding resistance to beta-lactams (*bla*_TEM-1_, two copies of *bla*_CTX-M-3_, and *bla*_AmpC_), aminoglycosides [*ant*(3′′)-I, aph(6)-Id, aph(3′′)-Ib, and aac(3)-II], macrolides [*mph*(2′)-I and *macA*], colistin (*arnA*), sulfonamide-trimethoprim (*sul1, sul2*, and *dfrA17*), and tetracycline [*tet*(A) and two copies of *tet*(C)]. Further analysis discovered mutations encoding amino acid substitutions at TCG(S)−83→TTG(L),GAC(D)−87→AAC(N) within the quinolone resistance-determining regions (QRDR) of *gyrA*, and AGC(S)−80→ATT(I) of *parC*. In addition, the genome encodes numerous transposases and insertion sequences, highlighting the importance of genomic exchange in creating the NB8 pathogenic phenotype.

The draft genome sequence of UPEC NB8 will aid in precise genetic manipulation and thereby further improve the study of UPEC virulence.

### Accession number(s).

The genome sequences of the uropathogenic *E. coli* NB8 have been deposited at DDBJ/EMBL/GenBank under the accession no. LBIS00000000.
